# MicroRNA mimicry blocks pulmonary fibrosis

**DOI:** 10.15252/emmm.201303604

**Published:** 2014-09-19

**Authors:** Rusty L Montgomery, Guoying Yu, Paul A Latimer, Christianna Stack, Kathryn Robinson, Christina M Dalby, Naftali Kaminski, Eva van Rooij

**Affiliations:** 1 miRagen Therapeutics, IncBoulder, CO, USA; 2 Section of Pulmonary, Critical Care and Sleep Medicine, Yale School of MedicineNew Haven, CT, USA; 3 Hubrecht Institute, KNAW and University Medical Center UtrechtUtrecht, The Netherlands

**Keywords:** microRNA, mimic, miR-29, pulmonary fibrosis, therapeutics

## Abstract

Over the last decade, great enthusiasm has evolved for microRNA (miRNA) therapeutics. Part of the excitement stems from the fact that a miRNA often regulates numerous related mRNAs. As such, modulation of a single miRNA allows for parallel regulation of multiple genes involved in a particular disease. While many studies have shown therapeutic efficacy using miRNA inhibitors, efforts to restore or increase the function of a miRNA have been lagging behind. The miR-29 family has gained a lot of attention for its clear function in tissue fibrosis. This fibroblast-enriched miRNA family is downregulated in fibrotic diseases which induces a coordinate increase of many extracellular matrix genes. Here, we show that intravenous injection of synthetic RNA duplexes can increase miR-29 levels *in vivo* for several days. Moreover, therapeutic delivery of these miR-29 mimics during bleomycin-induced pulmonary fibrosis restores endogenous miR-29 function whereby decreasing collagen expression and blocking and reversing pulmonary fibrosis. Our data support the feasibility of using miRNA mimics to therapeutically increase miRNAs and indicate miR-29 to be a potent therapeutic miRNA for treating pulmonary fibrosis.

## Introduction

Based on gain- or loss-of-function data collected in animal disease models using genetics or pharmacological modulation of microRNAs (miRNAs), it is now well accepted that miRNAs are important players during disease. These studies, combined with recent positive clinical efficacy data (Janssen *et al*, 2013), underscore the relevance of miRNAs and the viability for miRNAs to become the next class of therapeutics. Indeed, miRNAs have several advantages as therapeutic intervention points in that they are small and comprise a known sequence. Additionally, since a single miRNA can regulate numerous target mRNAs within biological pathways, modulation of a miRNA in principle allows for influencing an entire gene network and modifying complex disease phenotypes (van Rooij & Olson, 2012).

While many studies have shown therapeutic efficacy using single-stranded miRNA inhibitors called antimiRs, efforts to restore or increase the function of a miRNA have been lagging behind (van Rooij *et al*, 2012). Currently, miRNA function can be increased either by viral overexpression or by using synthetic double-stranded miRNAs. So far, the use of adeno-associated viruses (AAV) to drive expression of a given miRNA for restoring its activity *in vivo* has shown to be effective in a mouse model of hepatocellular and lung carcinoma (Kota *et al*, 2009; Kasinski & Slack, 2012) and spinal and bulbar muscular atrophy (Miyazaki *et al*, 2012), while the use of unformulated synthetic oligonucleotide-based approaches to increase miRNA levels has not been well explored.

The microRNA-29 (miR-29) family is well characterized for their ability to regulate extracellular matrix proteins (He *et al*, 2013). The family consists of miR-29a, miR-29b, and miR-29c, which are expressed as 2 bicistronic clusters (miR-29a/miR-29b-1 and miR-29b-2/miR-29c), and are largely homologous in sequence with only a few mismatches between the different members in the 3′ regions of the mature miRNA (van Rooij *et al*, 2008). All three members are reduced in different types of tissue fibrosis, and therapeutic benefit of increasing miR-29 levels has been shown for heart (van Rooij *et al*, 2008), kidney (Qin *et al*, 2011; Wang *et al*, 2012; Xiao *et al*, 2012), liver (Roderburg *et al*, 2011; Sekiya *et al*, 2011; Zhang *et al*, 2012), lung (Cushing *et al*, 2011; Xiao *et al*, 2012), and systemic sclerosis (Maurer *et al*, 2010).

Our data indicate that miRNA mimics with modifications for stability, and cellular uptake can be used to replicate endogenous functions of miR-29. Systemic delivery of synthetic miR-29b mimic increases miR-29b levels *in vivo* for several days without observable side effects or effects on gene expression. However, therapeutic treatment with miR-29b mimic in the setting of pulmonary fibrosis restores the bleomycin-induced reduction of miR-29 and blocks and reverses pulmonary fibrosis, which coincides with a repression of miR-29 target genes that are induced during the disease process.

Our data support the feasibility of using miRNA mimics to therapeutically increase miRNAs and indicate miR-29 to be a potent therapeutic miRNA as treatment for pulmonary fibrosis.

## Results and Discussion

### miR-29 mimicry *in vitro* and *in vivo*

**Figure 1:**
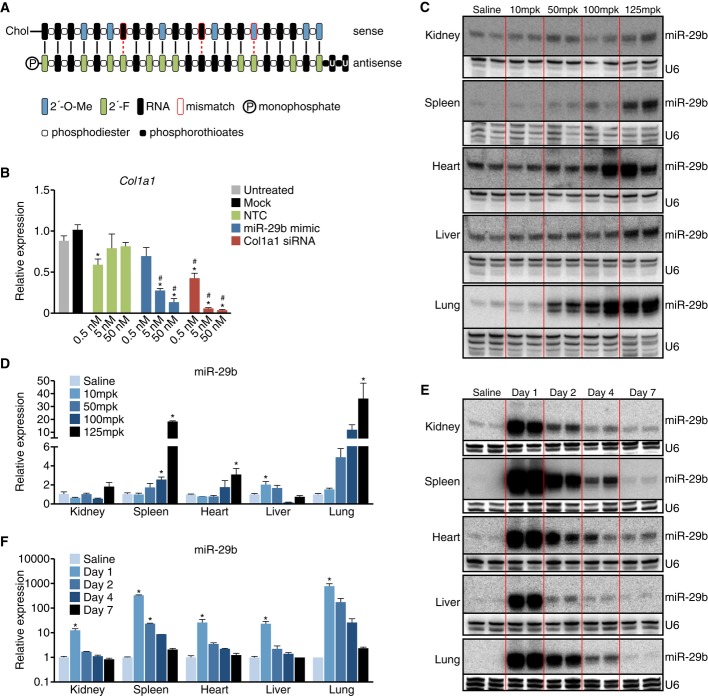
Pharmacokinetic properties of miR-29b mimic The double-stranded miR-29 mimics design contains a “guide strand” or “antisense strand” that is identical to the miR-29b, with a UU overhang on the 3′ end, modified to increase stability, and chemically phosphorylated on the 5′ end and a “passenger strand” or “sense strand” that contains 2′-O-Me modifications to prevent loading into RNA-induced silencing complex (RISC) as well as increase stability and is linked to cholesterol for enhanced cellular uptake. Several mismatches are introduced in the sense strand to prevent this strand from functioning as an antimiR.Transfection experiments in NIH 3T3 show a dose-dependent decrease in *Col1a1* with increasing amount of miR-29b mimic compared to either untreated or mock-treated cells. An siRNA directly targeting *Col1a1* was taken along as a positive control. *P < 0.05 versus mock, ^#^*P* < 0.05 versus untreated.Northern blot analysis for miR-29b in different tissues 4 days after intravenous injection with 10, 50, 100, or 125 mpk miR-29b mimic indicates delivery to all tissues at the highest dose, with the most effective delivery taking place to the lungs and spleen compared to saline-injected mice. U6 is used as a loading control.Real-time quantification of miR-29b mimicry indicates an increased level of miR-29b at the higher dose levels with the most efficient delivery to the lungs and spleen (*n* = 4 per group). *P < 0.05 versus saline-injected animals.Northern blot analysis for miR-29b in different tissues 1, 2, 4, and 7 days after intravenous injection with 125 mpk of mimic indicates the presence of miR-29b mimic in all tissues examined, with a longer detection in lung and spleen. U6 is used as a loading control.Real-time quantification of miR-29b mimicry indicates an increased level of miR-29b in all tissues measured which is maintained the longest in lungs and spleen (*n* = 4 per group). *P < 0.05 versus saline-injected animals.

Synthetic RNA duplexes can be used to therapeutically mimic or increase the level of a miRNA to enhance the endogenous activity of the miRNA of interest. These miRNA mimics harbor chemical modifications for stability and cellular uptake. We designed double-stranded miR-29 mimics utilizing lessons learned from antisense and siRNA technologies. The “guide strand” or “antisense strand” is identical to the miR-29b, with a UU overhang on the 3′ end, modified to increase stability, and chemically phosphorylated on the 5′ end. Since the guide strand has to function as a miRNA and the RISC machinery in the cell needs to recognize it as such, the allowed chemical modifications are limited. The 2′-F modification helps to protect against exonucleases, hence making the guide strand more stable, while it does not interfere with RISC loading. The “passenger strand” or the “sense strand” contains 2′-O-Me modifications to prevent loading into RNA-induced silencing complex (RISC) as well as increase stability and is linked to cholesterol for enhanced cellular uptake. Several mismatches are introduced to prevent this strand from functioning as an antimiR and to lessen hybridization affinity for the guide strand (Fig 1A).

To test for functional efficacy, we transfected miR-29b mimic into a mouse fibroblast cell line (NIH 3T3) and measured the effect on *Collagen1a1* (*Col1a1*) expression, a known direct target gene of miR-29 (van Rooij *et al*, 2008). Increasing amount of miR-29b mimic showed a dose-dependent decrease in *Col1a1*, compared to either untreated or control oligo treated cells, indicating the miR-29b mimic to be functional. An siRNA directly targeting *Col1a1* was taken along as a positive control (Fig 1B).

To start exploring the *in vivo* applicability and distribution of miR-29 mimic, we injected mice intravenously with 10, 50, 100, or 125 mg per kg (mpk) and sacrificed them 4 days later. Northern blot analysis on multiple tissues indicated little to no increase in miR-29b in kidney or liver samples compared to saline control. Cardiac distribution was detected; however, this appeared to be quite variable and spleen delivery could be observed at the highest dose only. In contrast, delivery to the lungs could be observed at all 3 of the highest doses 4 days after injection (Fig 1C). No effects on liver function (transaminase, ALT) were observed in the plasma, indicating that these miRNA mimics are well tolerated at these doses (Supplementary Fig S1). Real-time PCR demonstrated similar results with robust dose-dependent distribution of the miR-29b mimic to the lung compared to saline-injected animals (Fig 1D). Additionally, real-time PCR analysis of miR-29 targets showed no regulation at the mRNA level in the treated animals except for *Col3a1* at the highest dose in the spleen (Supplementary Fig S2). This suggests that the target genes are either at steady state in non-stressed animals and that mimics lower target genes when they are elevated, or that functional delivery was inadequate or insufficient.

To gain more insights into the *in vivo* stability of miRNA mimics, we injected 125 mpk of miR-29b mimic and sacrificed the mice 1, 2, 4, or 7 days later. Robust presence of miR-29b mimic could be detected by both Northern blot and real-time PCR analysis 1 day after injection in all tissues examined; however, tissue clearance greatly differed thereafter (Fig 1E and F). Liver and kidney rapidly cleared miR-29b mimic with minimal detection after day 1. Lung and spleen demonstrated the most pronounced detection of miR-29b mimic over time, which sustained at least 2–4 days post-treatment (Fig 1E and F). The increase was specific for miR-29b without any effect on miR-29a and miR-29c levels as measured by real-time PCR (Supplementary Fig S3). Also, here real-time PCR analysis of miR-29 targets showed no downregulation at the mRNA level in non-stressed animals (Supplementary Fig S4).

Together, these data indicate that unformulated miR-29b mimic can increase the miRNA level with tissue-dependent clearance and delivery efficiency, without any clear effect on gene expression under baseline conditions.

### miR-29b mimic blunts bleomycin-induced pulmonary fibrosis

Current treatments of tissue fibrosis mostly rely on targeting the inflammatory response; however, these are ultimately ineffective in preventing progression of the disease, underscoring the need for new mechanistic insights and therapeutic approaches (Friedman *et al*, 2013). Recent studies indicate the involvement of miRNAs in pulmonary fibrosis (Pandit *et al*, 2011).

**Figure 2:**
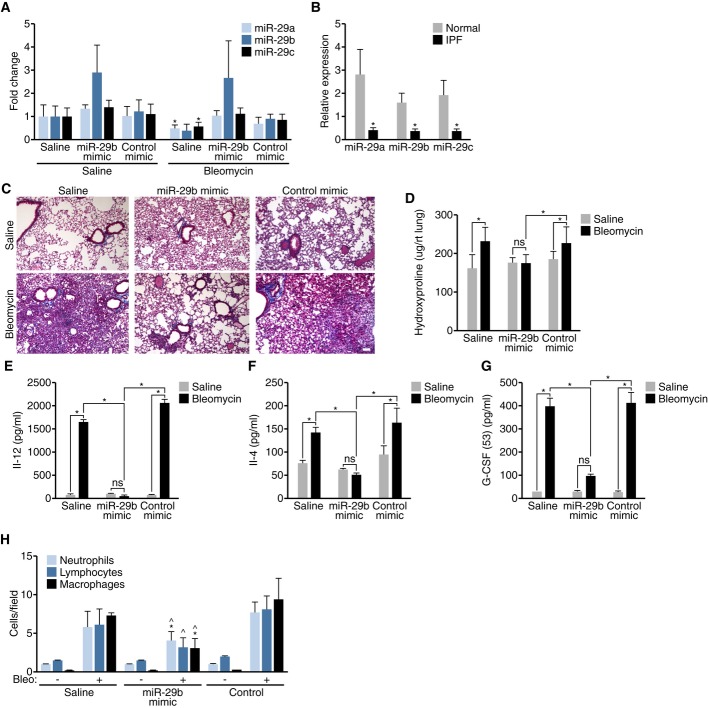
miR-29b mimic blunts pathological signs of bleomycin-induced pulmonary fibrosis A Real-time PCR analysis indicates a reduction in all miR-29 family members in response to bleomycin, while miR-29 mimic treatment resulted in the increased detection of miR-29b levels compared to either control- or saline-injected animals. *P < 0.05 versus Saline/Saline. B Real-time PCR analysis indicated a comparable decline in miR-29 levels in pulmonary biopsies of patients with idiopathic pulmonary fibrosis (IPF) compared to normal controls. *P < 0.05 versus Normal. C Histological examination by trichrome staining showing pronounced fibrotic and inflammatory response in response to bleomycin, which was blunted by miR-29b mimic treatment. Scale bar indicates 100 μm. D Hydroxyproline measurements to assay for total collagen content showed a significant increase following bleomycin treatment in both saline- and control-treated groups, while there was no statistical difference in the miR-29 mimic-treated group between saline- and bleomycin-treated mice. E–G Cytokine measurements on bronchoalveolar lavage (BAL) fluids indicated a significantly higher concentrations of IL-12 (E), IL-4 (F), and G-CSF (G) were detectable in BAL fluids from lungs from bleomycin-treated mice, which was reduced with miR-29b mimic (*n* = 4). *P < 0.05. H Bleomycin treatment increases the detection of immune cells in BAL fluids which was significantly reduced in the presence of miR-29b mimic, while the control mimic had no effect (*n* = 4)., *P < 0.05 versus Saline/Bleo, ^^^*P* < 0.05 versus Control/Bleo.

Due to the preferential lung distribution of our mimic, we set out to explore whether stress and subsequent induction of target gene expression would allow for detectable changes in mRNA target genes and downstream therapeutic effects in response to miR-29b mimic. To this end, we used the bleomycin-induced model of pulmonary fibrosis as described (Pandit *et al*, 2010) and injected the mice with 100 mpk miR-29b mimic, control or a comparable volume of saline at two time-points: 3 and 10 days after bleomycin treatment. As expected, 14 days after bleomycin treatment, miR-29 levels were reduced, while miR-29b mimic treatment resulted in the increased detection of miR-29b levels compared to either control or saline-injected animals as measured by real-time PCR, albeit with a high level of variation (Fig 2A). It is currently unclear why the increase in miR-29b levels is less than we detected in baseline mice (Fig 1). A comparable decline in miR-29 levels was observed in pulmonary biopsies of patients with idiopathic pulmonary fibrosis (IPF) compared to normal controls (Fig 2B). Histological examination by trichrome staining showed a clear and robust fibrotic and inflammatory reaction in response to bleomycin, which was blunted by miR-29b mimic treatment (Fig 2C). Additionally, hydroxyproline measurements to assay for total collagen content indicated a significant increase following bleomycin treatment in both saline and control-treated groups, while there was no statistical difference in the miR-29 mimic-treated group between saline and bleomycin-treated mice, indicating that miR-29b mimic treatment blunts bleomycin-induced pulmonary fibrosis (Fig 2D).

Innate immune effector signaling pathways act as important drivers of myofibroblast transdifferentiation by provoking fibrosis. To further characterize the therapeutic effects of miR-29b mimic in the setting of bleomycin-induced pulmonary fibrosis, we performed bronchoalveolar lavage (BAL) on these mice and assessed cytokine levels. Significantly higher concentrations of IL-12, IL-4, and G-CSF were detectable in BAL fluids from lungs from bleomycin-treated mice, which were reduced with miR-29b mimic (Fig 2E–G). Additionally, the bleomycin-induced elevation of detectable immune cells in BAL fluids was significantly reduced in the presence of miR-29b mimic (Fig 2H), indicating an inhibitory effect on the immune response by miR-29b, which is likely secondary to the antifibrotic-effect. To determine if miR-29 mimicry has a direct effect on macrophages, we transfected miR-29b mimic and control into macrophage cells, RAW 264.7, and harvested the supernatant at 24 and 48 h after transfection. IFN-r, IL-1B, IL-2, IL-4, IL-5, IL-6, KC, IL-10, IL-12P70, and TNF-α were assessed, with no significant differences observed between miR-29b mimic and control (P. Latimer and R. Montgomery, unpublished data). By real-time PCR analysis, there were no significant differences in *Tgfb1, Ctgf, FGF1,* or *PDGF* expression; however, we did observe a significant difference in *Csf3, Igf1*, and *Kc* expression (Supplementary Fig S5 and P. Latimer and R. Montgomery, unpublished data).

**Figure 3:**
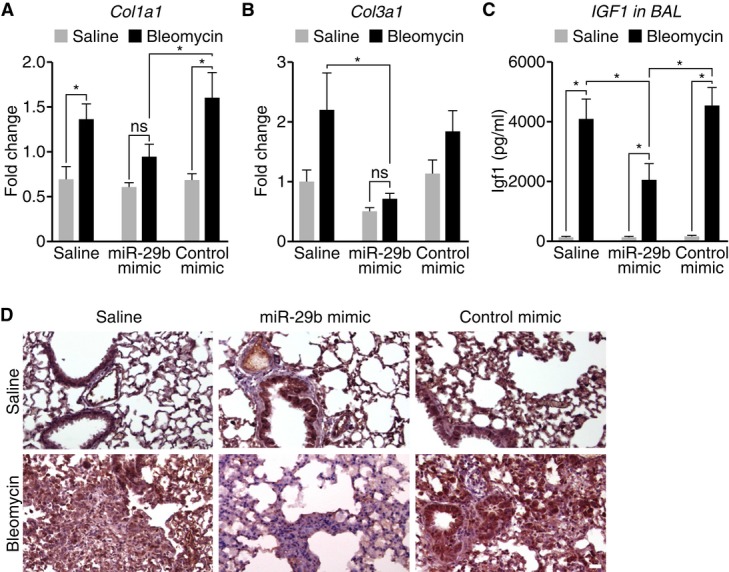
*In vivo* mimicry of miR-29b represses the induction of miR-29 target genes during pulmonary fibrosis A, B Bleomycin treatment increases the expression of *Col1a1* (A) and *Col3a1* (B), and the presence of miR-29b mimic inhibits *Col1a1* and *Col3a1* as measured by real-time PCR. MiR-29b mimicry has no effect on target repression under baseline conditions. (*n* = 6–8), *P < 0.05. C IGF1 levels in BAL fluids increase following bleomycin treatment which were significantly blunted in the presence of miR-29 mimic compared to both saline- and control mimic-treated mice (*n* = 4). *P < 0.05. D Immunohistochemistry demonstrated robust detection of IGF1 after bleomycin treatment, which was reduced in the miR-29b mimic-treated group compared to saline- or control mimic-treated mice. Scale bar indicates 50 μm.

Since it has been well validated that miR-29 functions through the regulation of many different extracellular matrix related genes (van Rooij & Olson, 2012), we confirmed the regulation of a subset of these target genes. While a significant increase in *Col1a1* and a trend increase in *Col3a1* expression were observed with bleomycin treatment in both saline and control-treated groups, the detection of *Col1a1* and *Col3a1* was significantly blunted in the presence of miR-29b mimic in the bleomycin-treated mice (Fig 3A and B). Interestingly, the increase in Igf1 levels in BAL fluids following bleomycin treatment was significantly blunted in the presence of miR-29 mimic compared to both saline and control-treated mice (Fig 3C). Furthermore, immunohistochemistry for Igf1 demonstrated robust reductions in Igf1 after bleomycin in miR-29b mimic-treated groups compared to saline or controls (Fig 3D).

**Figure 4:**
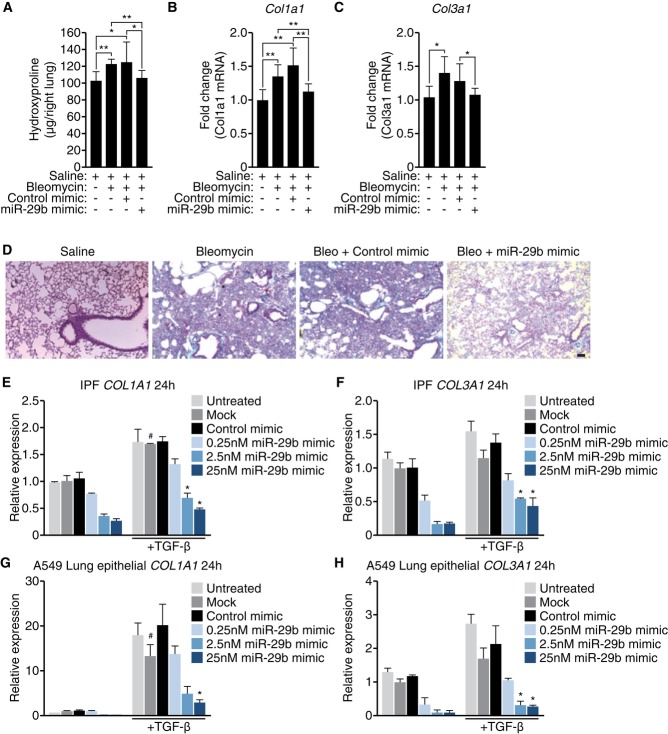
Therapeutic mimicry of miR-29 attenuates bleomycin-induced fibrosis A Hydroxyproline assessment showed a significant increase following bleomycin treatment in both saline- and control-treated groups; however, there was no statistical difference in the miR-29 mimic-treated group between saline- and bleomycin-treated mice. *P < 0.05 (*n* = 8). B, C Real-time PCR analysis showed a significant increase of *Col1a1* (B) and *Col3a1* (C) after bleomycin treatment. miR-29b mimic treatment normalized both *Col1a1* and *Col3a1* to vehicle-treated expression levels. *P < 0.05 (*n* = 8). D Histological examination by trichrome staining showing robust fibrosis in response to bleomycin, which was blunted by miR-29b mimic treatment. Scale bar indicates 50 μm. E, F Primary pulmonary fibroblasts from patients with IPF were treated with vehicle or TGF-β and transfected with control mimic or miR-29b mimic. Real-time PCR was performed for *Col1a1* (E) and *Col3a1* (F). miR-29b mimic treatment showed a dose-dependent reduction in both collagens. G, H A549 cells were treated with vehicle or TGF-β and transfected with control mimic or miR-29b mimic. Real-time PCR was performed for *Col1a1* (G) and *Col3a1* (H). miR-29b mimic treatment showed a dose-dependent reduction in expression of both *Col1a1* and *Col3a1*.

After establishing that early (days 3 and 10) miR-29 mimicry was sufficient to prevent bleomycin-induced fibrosis, we sought to determine if miR-29 mimicry affects established fibrosis. For that purpose, we started the miR-29b mimic administration at day 10 post-bleomycin and repeated the doses at days 14 and 17 after which we harvested the lungs at day 21. Hydroxyproline assessment of the right lung showed a significant increase with bleomycin in both saline and control-treated lungs; however miR-29b mimic treatment blunted this effect (Fig 4A). Furthermore, bleomycin treatment resulted in significant increases in *Col1a1* and *Col3a1* expression, which was also normalized with miR-29b mimic treatment (Fig 4B and C). Histological assessment by trichrome staining corroborated this effect, whereby bleomycin induced significant fibrosis with saline or control treatment which was blunted with miR-29b mimicry (Fig 4D).

While we believe these effects are mediated through regulation of collagen production from lung fibroblasts, we are not able to rule out effects from other collagen producing cells. To address this, we assessed miR-29b mimic effects *in vitro* from different lung cells, including primary fibroblasts from IPF patients and A549 cells, a lung epithelial cell line. As expected, primary pulmonary fibroblasts from IPF patients show an increase in *Col1a1* and *Col3a1* in response to TGF-β (Fig 4E and F). This effect was dose-dependently blunted with miR-29b mimic treatment at both 24 and 48 h (Fig 4E and F and P. Latimer and R. Montgomery, unpublished data). Similarly, A549 cells respond to TGF-β with robust increases in *Col1a1*and *Col3a1* expression (Fig 4G and H). Again, miR-29b mimic treatment is able to block collagen induction, in both TGF-β treated as well as baseline conditions (Fig 4G and H). The effects on collagen induction are much more robust in the A549 cells compared to primary IPF cells; however, this is likely due to the already high expression in primary fibroblasts from IPF patients. While A549 cells are epithelial cells and the contribution of these cells to pulmonary fibrosis is still debated, these data do show that miR-29 mimicry can also block collagen induction in this cell type. Additionally, we looked at miR-29 effects in the macrophage line, Thp-1, but could not observe collagen expression in the cells, regardless of stimulation (P. Latimer and R. Montgomery, unpublished data). These data suggest miR-29b mimicry is able to blunt collagen-induced expression in fibroblasts and epithelial cells. These data are all in line with a recent paper by Xiao *et al* (2012), in which they showed that gene transfer of miR-29 using a Sleeping Beauty-transposon system was capable of preventing and treating bleomycin-induced pulmonary fibrosis, further underscoring the therapeutic potential for increasing miR-29.

Our data suggests the feasibility of using microRNA mimics to restore the function of lost or downregulated miRNAs. However, it is important to note that because RISC incorporation is required for appropriate miRNA function, the allowed chemical modifications are limited; thus, miRNA mimics are far less stable than antimiR chemistries, and dosage and administration regiments need to be worked out in detail as the doses used in most animal studies to date are probably significantly higher than what would be therapeutically acceptable. Another potential issue with miRNA replacement therapies is the challenge of restoring the level of a downregulated miRNA while preventing the introduction of supraphysiological levels of the miRNA. Additionally, although a miRNA mimic can have therapeutic use, potential off target effects of miRNA mimicry can occur as a result of delivery to tissues or cells that do not normally contain the miRNA of interest. Thus, targeting those patients with exceedingly low levels of the miRNA and delivery to the appropriate cell type or tissue are important aspects of effective miRNA mimicry. In the case of pulmonary fibrosis, this may suggest that direct delivery through the inhaled route may be an appealing alternative to traditional routes of administration. Lastly, it should also be noted that double-stranded miRNA mimics can potentially induce a non-specific interferon response through toll-like receptors (Peacock *et al*, 2011), and thus careful assessment of dosing and off target effects will be required.

Despite the fact that the currently available mimic chemistries require further optimization to increase stability and efficacy, our data clearly support the notion that miRNA mimics can be used to therapeutically increase miRNA levels and that miR-29 is a potent therapeutic miRNA for treating pulmonary fibrosis. Validating these data in additional models of pulmonary fibrosis will be important before we can translate these data into a clinical setting. The lack of observable effects on gene expression under baseline conditions might relieve some of the concerns regarding the systemic gene regulatory effects of miRNA mimics. Our data, combined with the fact that the first synthetic formulated miRNA mimic for miR-34 is currently entering a Phase 1 trial in patients with primary liver cancer (Bouchie, 2013), provides great promise for mimics as novel miRNA therapeutics.

## Materials and Methods

### Animals

All animal studies were reviewed and approved by the Animal Care and Use Committee (IACUC) at miRagen Therapeutics, Inc. (murine pharmacokinetic studies) or the University of Pittsburgh IACUC (bleomycin study) and comply with Federal and State guidelines concerning the use of animals in research and teaching as defined by The Guide For the Care and Use of Laboratory Animals (NIH Publication No. 80-23, revised 1985).

For the pharmacokinetic studies, *n* = 40 C57Bl/6 male mice of 7–8 weeks of age were used (Harlan). For the bleomycin studies, *n* = 200 C57Bl/6 female mice of 8–11 weeks of age were used (Toconic lab).

### Synthesis and delivery of oligonucleotide chemistries

The oligonucleotides were synthesized at miRagen Therapeutics, Inc. utilizing standard phosphoramidite solid phase synthesis. The sense strands of both the miR-29b mimic and the non-targeting control mimic were conjugated with cholesterol at the 3′ end. miRNA mimic duplexes were annealed at equimolar concentrations, heated to 95°C, and then cooled to room temperature. The control duplex sequence does not target any known murine or human transcripts by BLAST analysis. Unless else indicated, *in vivo* delivery of the oligonucleotide chemistries was achieved by low pressure intravenous (i.v.) injections via the tail vein of either adult male C56Bl6 mice (Harlan, Indianapolis). All chemistries were dissolved and injected in a comparable end volume of saline after which the animals were examined for obvious side effects of the chemistries. Tissue samples were collected at the indicated timepoints for molecular or histological examination.

### *In vitro* experiments

NIH 3T3 cells were purchased from ATCC and cultured in Dulbecco's Minimum Essential Media (high glucose), (Hyclone) supplemented with 4 mM l-Glutamine, 1 mM Sodium Pyruvate, and 10% BCS (CO serum company). Cells were transfected with 0.2 μl/well (96 well) Dharmafect I (Thermofisher Scientific) as per the manufacturers' instructions. Cells were harvested 48 h after transfection, and gene expression was analyzed using qPCR (Life Tech).

A549 cells (ATCC® CCL-185™) were maintained in F-12K Medium (ATCC® Catalog No. 30-2004) with 10% FBS and kept in a 37°C incubator with a 5% CO_2_ air atmosphere. Cells were transfected with 0.2 μl/well Dharmafect I (Thermofisher Scientific) as per the manufacturers' protocol. *TGF-β* was added at the time of transfection. Cells were harvested 24 and 48 h post-transfection, and RNA expression was analyzed using qPCR (Life Technologies).

LL 29 (AnHa) (ATCC ® CCL-134™) cells were maintained in Ham's F12K medium with 15% FBS and kept in a 37°C incubator with a 5% CO_2_ air atmosphere. Cells were transfected with 0.2 μl/well Dharmafect I (Thermofisher Scientific) as per the manufacturers' protocol. *TGF-β* was added at the time of transfection. Cells were harvested 24 and 48 h post-transfection, and RNA expression was analyzed using qPCR (Life Technologies).

Col1a1 Sense Strand: 5′- GCAAGACAGUCAUCGAAUA Col1a1 Antisense Strand: 3′- CGUUCUGUCAGUAGCUUAU

### Real-time PCR

For *in vivo* real-time PCR analysis, RNA was extracted from cardiac tissue using Trizol (Invitrogen) after which 2 μg RNA from each tissue sample was used to generate cDNA using Super Script II reverse transcriptase per manufacturer's specifications (Invitrogen). Taqman MicroRNA assay (Applied Biosystems, ABI) was used to detect changes in miRNAs or genes according the manufacturer's recommendations, using 10–100 ng of total RNA. U6 was used a control for miRNA analysis, and Gapdh was used as a control for gene analysis.

### Northern blotting

Total RNA was isolated from cardiac tissue samples by using Trizol reagent (Gibco/BRL). Northern blots (van Rooij *et al*, 2008) to detect microRNAs were performed as described previously described. A U6 probe served as a loading control (IDT). 10 ug of total RNA from the indicated tissues was loaded on 20% acrylamide denaturing gels and transferred to Zeta-probe GT genomic blotting membranes (Bio-Rad) by electrophoresis. After transfer, the blots were cross-linked and baked at 80°C for 1 h. To maximize the sensitivity of miRNA detection, oligonucleotide probes were labeled with the Starfire Oligos Kit (IDT, Coralville, IA) and α-^32^P dATP (Amersham or Perkin Elmer). Probes were hybridized to the membranes overnight at 39°C in Rapid-hyb buffer (Amersham), after which they were washed twice for 10 min at 39°C with 0.5× SSC containing 0.1% SDS. The blots were exposed and quantified by PhosphorImager analysis (GE HealthCare Life Sciences) and a U6 probe served as a loading control (ABI). The intensity of the radioactive signal was used to quantify the fold change in expression using a phosphorimager and ImageQuant (Bio-Rad).

### Bleomycin model for pulmonary fibrosis

Mice were anesthetized by placing them in a chamber having paper towels soaked with 40% isoflurane solution. 0.0375 U of bleomycin (Hospira, IL) was administered intratracheally in 50 μl of 0.9% saline. Mimicry of miR-29b and scramble miR-29b were administrated at dose of 100 mpk in tail with the control of 0.9% saline. To determine that miR-29 mimicry could affect early fibrosis, we administered the mimic at days 3 and 10 after bleomycin treatment and sacrificed the lungs at day 14. To demonstrate that mimicry was effective against established fibrosis, we administered the miR-29b mimic at days 10, 14, and 17 after bleomycin or saline and sacrificed the mice at day 21. In both protocols, we harvested the lungs for histological analysis, hydroxyproline assay, and RNA extraction.

**The paper explained**

**Problem**

MicroRNAs (miRNAs) are important regulator of gene expression during disease. Over the last decade, great enthusiasm has evolved for microRNA (miRNA) therapeutics. However, while many studies have shown therapeutic efficacy using miRNA inhibitors, efforts to restore or increase the function of a miRNA have been lagging behind.

**Results**

The miR-29 family is a fibroblast-enriched miRNA family that is downregulated in fibrotic diseases whereby leading to a coordinate increase of many extracellular matrix genes. Here, we show that intravenous injection of synthetic RNA duplexes can increase miR-29 levels *in vivo* for several days. Moreover, therapeutic delivery of these miR-29 mimics during bleomycin-induced pulmonary fibrosis restores endogenous miR-29 function whereby decreasing collagen expression and blocking and reversing pulmonary fibrosis.

**Impact**

Our data provide great promise for the use of miRNA mimics to therapeutically increase miRNA levels *in vivo* and indicate miR-29 to be a potent therapeutic miRNA for treating pulmonary fibrosis.

### Idiopathic pulmonary fibrosis samples

De-identified lung tissue samples were obtained through the University of Pittsburgh Health Sciences Tissue Bank. Sixteen IPF lung tissue samples were obtained from surgical remnants of biopsies or lungs explanted from patients with IPF who underwent pulmonary transplantation. All of the experiments have been approved by the institutional Review Board at the University of Pittsburgh. The experiments conformed to the principles set out in the WMA Declaration of Helsinki (http://www.wma.net/en/30publications/10policies/b3/) and the NIH Belmont Report (http://www.hhs.gov/ohrp/humansubjects/guidance/belmont.html).

### Histology

Tissue sections (4 μm) were stained with Masson Trichrome (collagen/connective tissue), two slices per animal, two animals per group. Immune staining was performed after paraffin removal, hydration, and blocking, following the recommendation of the manufacturer (ABC detection system form Vector's lab, USA). Sections were incubated overnight at 4°C with the primary antibody (Igf1, diluted 1:100 in PBS) and during 1 h at room temperature with the secondary antibodies (Invitrogen, USA). The sections were counterstained with hematoxylin. The primary antibody was replaced by non-immune serum for negative controls. Finally, sections were mounted with mounting medium (DAKO, USA) and analyzed using a Nikon microscope.

### Hydroxyproline assay

Lung hydroxyproline was analyzed with hydroxyproline colorimetric assay kit from Biovision (Milpitas, CA) following manufacturer's instruction. Briefly, the lungs from control and experimental mice were dried until constant weight and hydrolyzed in 12 N HCl for 3 h at 120°C. The digestions reacted with Chloramine T reagent and visualized in DMAB reagent. The absorbance was measured at 560 nm in a microplate reader. Data are expressed as μg of hydroxyproline/right lung.

### Determination cytokines/chemokines

A human Cytokine/Chemokine Panel from Bio-Rad was used for simultaneous detection of analytes. The entire procedure was performed following manufacturer's instruction. Briefly, BALs were diluted fivefold, and assay was performed in 96-well filter plates. For the detection step, samples were incubated for 30 min with streptavidin conjugated to R-phycoerythrin and analyzed in the Bio-Plex suspension array system (Bio-Rad). Raw data were analyzed using Bioplex Manager software 6.0 (Bio-Rad). The cytokine standards supplied by the manufacturer were used to calculate the concentrations of the samples. The analytes that were below the detection range were not included in date interpretation. Also, samples that had a particular analyte below the detection range were excluded while calculating the median value.

### Statistical analysis

One-way ANOVA and Newman–Keuls multiple comparison post-test or a *t*-test were used to determine significance. *P *<* *0.05 was considered statistically significant. The exact *P*-values for each figure can be found in Supplementary Table S1.

## For more information

http://www.miragentherapeutics.com

http://medicine.yale.edu/lab/kaminski

http://www.hubrecht.eu/research/vanrooij/index.html

http://www.mirnatherapeutics.com
