# Supplementary material for: MicroRNA mimicry blocks pulmonary fibrosis
Source: EMBO Mol Med. 2014 Sep 19;6(10):1347–56. doi: 10.15252/emmm.201303604 (PMC4287936; doi:10.15252/emmm.201303604)
Supplement: Supplementary file 1 [file emmm0006-1347-sd1.pdf]

MicroRNA mimicry blocks pulmonary fibrosis

Rusty L. Montgomery[1#], Guoying Yu[2#], Paul A. Latimer[1], Chistianna Stack[1], Kathryn Robinson[1], Christina M. Dalby[1], Naftali Kaminski[2*], Eva van Rooij[1,3*]

[1] miRagen Therapeutics, Inc, 6200 Lookout Rd, Boulder, CO 80301, USA.

[2] Section of Pulmonary, Critical Care and Sleep Medicine, Yale School of Medicine, New Haven, CT 06520, USA.

[3] Hubrecht Institute, KNAW and University Medical Center Utrecht, 3584CT Utrecht, the Netherlands.

Supplementary Figure legends

Supporting information

Supplemental Figure S1. miR-29b mimic does not induce general signs of toxicity

MiR-29b mimic treatment does not induce any overt signs of liver or kidney toxicity as indicated by the lack of change in aspartate or alanine transaminases (AST and ALT). n=4 per group

Supplemental Figure S2. Increasing doses of miR-29b mimic fail to induce overt changes in gene expression under baseline conditions

Real-time PCR analysis indicates there to be no significant changes in expression in the different tissue 4 days after treatment with increasing doses of miR-29b mimic for *Col1a1* and *Col3a1* compared to Saline injected mice. n=4 per group, * $p<0.05$ compared to Saline injected.

Supplemental Figure S3. miR-29b mimic specifically increases miR-29b

MiR-29b mimicry specifically increases the level of miR-29b without affecting the level of miR-29a or miR-29c compared to Saline injected mice. The increase in miR-29c at day 1 might be due to some cross-reactivity of the real-time probe. n=4 per group

Supplemental Figure S4. miR-29b mimic does not induce any target changes baseline conditions in time

Real-time PCR analysis showed the absence of significant target changes at the indicated timepoints after injecting 125 mpk of miR-29b mimic for *Col1a1* and *Col3a1* compared to Saline injected mice. n=4 per group

Supplemental Figure S5. miR-29b mimic effects on gene expression in RAW cells.

Real-time PCR analysis showed significant increases in *Csf3*, *Igf1*, and *Kc* expression after miR-29b mimic treatment compared to vehicle or control mimic. * $p<0.05$ compared to Vehicle injected

Supplemental Table SI. P-values for each figure.

Figure	Comparison	p-value	Statistical analysis	n
1B	Mock vs. 0.5nM NTC	0,0114	ANOVA; Tukey multiple comparison	3
1B	Mock vs. 0.5nM miR-29	ns	ANOVA; Tukey multiple comparison	3
1B	Mock vs. 5.0nM miR-29	0,0001	ANOVA; Tukey multiple comparison	3
1B	Mock vs. 50nM miR-29	0,00001	ANOVA; Tukey multiple comparison	3
1B	Mock vs. 0.5 siRNA	0,0001	ANOVA; Tukey multiple comparison	3
1B	Mock vs. 5.0 siRNA	<0.0001	ANOVA; Tukey multiple comparison	3
1B	Mock vs. 50 siRNA	0,0001	ANOVA; Tukey multiple comparison	3
1B	Untreated vs. 5.0nM miR-29	0,0001	ANOVA; Tukey multiple comparison	3
1B	Untreated vs. 50nM miR-29	<0.0001	ANOVA; Tukey multiple comparison	3
1B	Untreated vs. 0.5 siRNA	0,0068	ANOVA; Tukey multiple comparison	3
1B	Untreated vs. 5.0 siRNA	<0.0001	ANOVA; Tukey multiple comparison	3
1B	Untreated vs. 50 siRNA	<0.0001	ANOVA; Tukey multiple comparison	3
1D Kidney	Saline vs. 125mpk	ns	ANOVA; Tukey multiple comparison	2-4
1D Spleen	Saline vs. 100mpk	0,0103	ANOVA; Tukey multiple comparison	2-4
1D Spleen	Saline vs. 125mpk	<0.0001	ANOVA; Tukey multiple comparison	2-4
1D Heart	Saline vs. 125mpk	0,0031	ANOVA; Tukey multiple comparison	2-4
1D Liver	Saline vs. 10mpk	0,0343	ANOVA; Tukey multiple comparison	2-4
1D Liver	Saline vs. 100mpk	ns	ANOVA; Tukey multiple comparison	2-4
1D Lung	Saline vs. 125mpk	0,0001	ANOVA; Tukey multiple comparison	2-4
1F Kidney	Saline vs. Day 1	0,0028	ANOVA; Tukey multiple comparison	2
1F Spleen	Saline vs. Day 1	<0.0001	ANOVA; Tukey multiple comparison	2
1F Spleen	Saline vs. Day 2	0,0338	ANOVA; Tukey multiple comparison	2
1F Heart	Saline vs. Day 1	0,0452	ANOVA; Tukey multiple comparison	2
1F Liver	Saline vs. Day 1	0,0245	ANOVA; Tukey multiple comparison	2
1F Lung	Saline vs. Day 1	0,0196	ANOVA; Tukey multiple comparison	2
2A	miR-29a: Saline bleomycin vs saline saline	2,30E-03	Fisher T-Test	8
2A	miR-29c: Saline bleomycin vs saline saline	1,70E-02	Fisher T-Test	8
2B	miR-29a: IPF vs Normal	1,45E-06	Fisher T-Test	17
2B	miR-29b: IPF vs Normal	2,62E-05	Fisher T-Test	17
2B	miR-29c: IPF vs Normal	4,81E-05	Fisher T-Test	17
2D saline	bleomycin vs saline	6,84E-03	Fisher T-Test	8
2D control mimic	bleomycin vs saline	3,75E-02	Fisher T-Test	8
2D bleomycin	control mimic vs mimic	1,44E-02	Fisher T-Test	8
2E saline	bleomycin vs saline	1,24E-04	Fisher T-Test	6
2E control mimic	bleomycin vs saline	3,45E-05	Fisher T-Test	6
2E bleomycin	mimic vs saline	2,13E-06	Fisher T-Test	6
2E bleomycin	mimic control vs mimic	1,45E-07	Fisher T-Test	6
2F saline	bleomycin vs saline	2,30E-03	Fisher T-Test	6
2F control mimic	bleomycin vs saline	1,67E-02	Fisher T-Test	6
2F bleomycin	mimic vs saline	1,40E-03	Fisher T-Test	6

2E bleomyciF	mimic control vs mimic	1,76E-03	Fisher T-Test	6
2G saline	bleomycin vs saline	4,52E-04	Fisher T-Test	6
2G control mimic	bleomycin vs saline	1,12E-06	Fisher T-Test	6
2G bleomycin	mimic vs saline	4,52E-03	Fisher T-Test	6
2G bleomycin	mimic control vs mimic	3,61E-03	Fisher T-Test	6
2H	Neutrophils: mimic vs saline/bleo	2,38E-02	Fisher T-Test	4
2H	Lymphocytes: mimic vs saline/bleo	3,72E-02	Fisher T-Test	4
2H	Macrophages: mimic vs saline/bleo	3,83E-02	Fisher T-Test	4
2H	Neutrophils: mimic vs control/bleo	3,40E-03	Fisher T-Test	4
2H	Lymphocytes: mimic vs control/bleo	1,66E-03	Fisher T-Test	4
2H	Macrophages: mimic vs control/bleo	4,87E-04	Fisher T-Test	4
3A saline	saline vs bleomycin	4,14E-04	Fisher T-Test	8
3A control mimic	saline vs Bleomycin	1,81E-03	Fisher T-Test	8
3A bleomycin	mimic vs mimic control	2,13E-02	Fisher T-Test	8
3B bleomycin	saline vs mimic	1,42E-02	Fisher T-Test	8
3C saline	saline vs Bleomycin	3,58E-04	Fisher T-Test	4
3C mimic	saline vs bleomycin	1,34E-03	Fisher T-Test	4
3C control mimic	saline vs bleomycin	4,24E-05	Fisher T-Test	4
3C bleomycin	saline vs mimic	2,03E-02	Fisher T-Test	4
3C bleomycin	mimic vs mimic control	3,66E-03	Fisher T-Test	4
4A	saline vs Bleo	2,39E-03	Fisher T-Test	8
4A	saline vs mimic control/bleo	3,27E-02	Fisher T-Test	8
4A	bleo vs mimic/bleo	2,64E-04	Fisher T-Test	8
4A	Mimic control/bleo vs mimic/bleo	4,51E-02	Fisher T-Test	8
4B	Saline vs Bleo	3,74E-04	Fisher T-Test	8
4B	Saline vs mimic control/bleo	2,26E-04	Fisher T-Test	8
4B	bleo vs mimic/bleo	7,23E-04	Fisher T-Test	8
4B	Mimic control/bleo vs mimic/bleo	4,06E-04	Fisher T-Test	8
4C	Saline vs bleo	4,13E-02	Fisher T-Test	8
4C	Mimic control/bleo vs mimic/Bleo	3,77E-02	Fisher T-Test	8
4E	+TGFB Mock vs +TGFB 2.5mpk miR-29	<0.0001	ANOVA; Tukey multiple comparison	3
4E	+TGFB Mock vs +TGFB 25mpk miR-29	<0.0001	ANOVA; Tukey multiple comparison	3
4E	+TGFB Mock vs Mock	0,0015	ANOVA; Tukey multiple comparison	3
4F	+TGFB Mock vs +TGFB 2.5mpk miR-29	0,0093	ANOVA; Tukey multiple comparison	3
4F	+TGFB Mock vs +TGFB 25mpk miR-29	0,0015	ANOVA; Tukey multiple comparison	3
4G	+TGFB Mock vs +TGFB 2.5mpk miR-29	ns	ANOVA; Tukey multiple comparison	3

4G	+TGFB Mock vs +TGFB 25mpk miR-29	0,0072	ANOVA; Tukey multiple comparison	3
4G	+TGFB Mock vs Mock	0,0011	ANOVA; Tukey multiple comparison	3
4H	+TGFB Mock vs +TGFB 0.25mpk miR-29	ns	ANOVA; Tukey multiple comparison	3
4H	+TGFB Mock vs +TGFB 2.5mpk miR-29	0.0070	ANOVA; Tukey multiple comparison	3
4H	+TGFB Mock vs +TGFB 25mpk miR-29	0,0052	ANOVA; Tukey multiple comparison	3

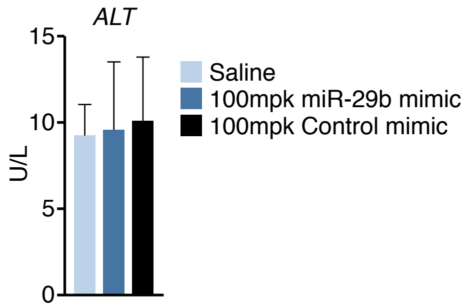
ALT
15
10
5
0
U/L
Saline
100mpk miR-29b mimic
100mpk Control mimic

Supp Fig 1

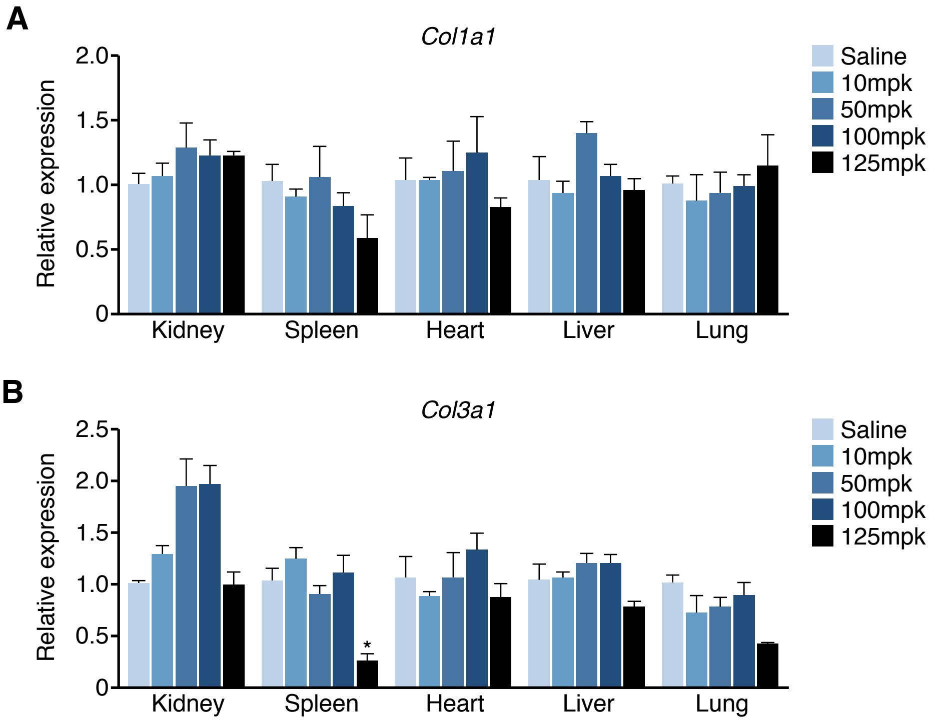
A
Col1a1
Relative expression
2.0
1.5
1.0
0.5
0
Kidney
Spleen
Heart
Liver
Lung
Saline
10mpk
50mpk
100mpk
125mpk
B
Col3a1
Relative expression
2.5
2.0
1.5
1.0
0.5
0
*
Kidney
Spleen
Heart
Liver
Lung
Saline
10mpk
50mpk
100mpk
125mpk

Supp Fig 2

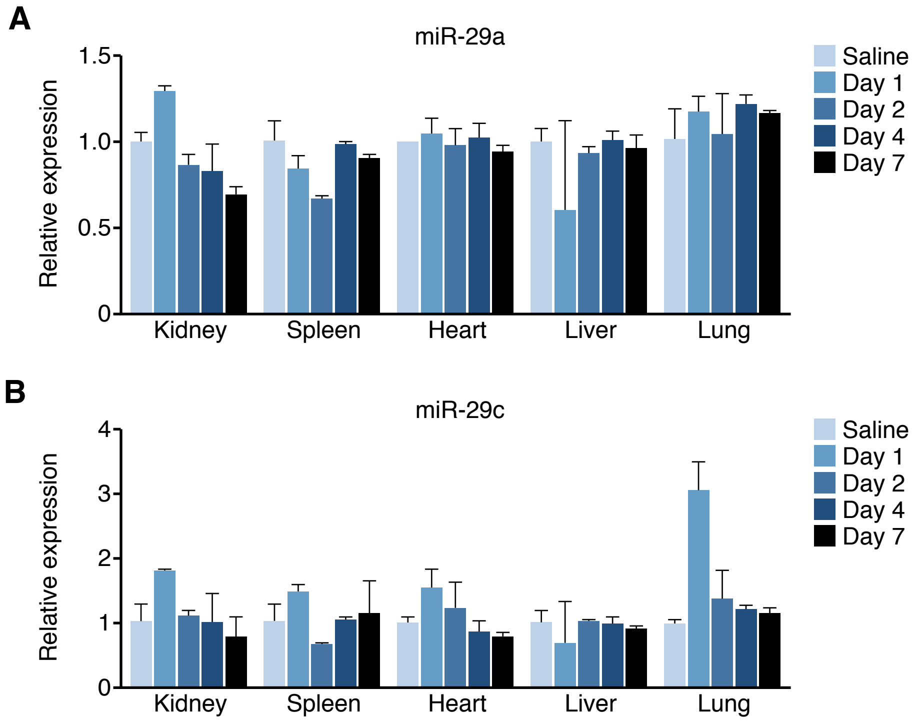
A
miR-29a
Relative expression
1.5
1.0
0.5
0
Kidney
Spleen
Heart
Liver
Lung
Saline
Day 1
Day 2
Day 4
Day 7
B
miR-29c
Relative expression
4
3
2
1
0
Kidney
Spleen
Heart
Liver
Lung
Saline
Day 1
Day 2
Day 4
Day 7

Supp Fig 3

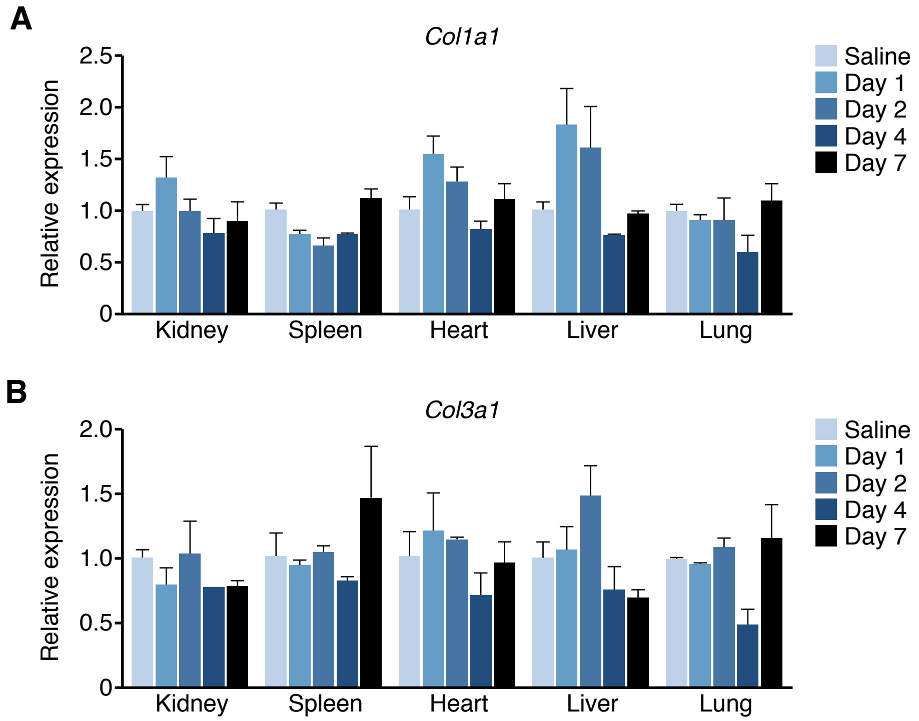
A
Col1a1
Relative expression
2.5
2.0
1.5
1.0
0.5
0
Kidney
Spleen
Heart
Liver
Lung
Saline
Day 1
Day 2
Day 4
Day 7
B
Col3a1
Relative expression
2.0
1.5
1.0
0.5
0
Kidney
Spleen
Heart
Liver
Lung
Saline
Day 1
Day 2
Day 4
Day 7

Supp Fig 4

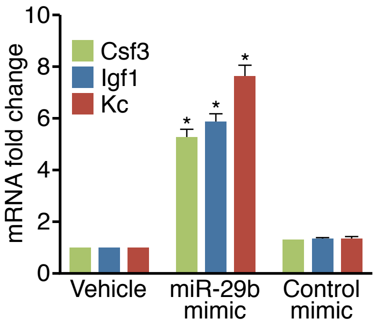

10
8
6
4
2
0
mRNA fold change
Csf3
Igf1
Kc
*
*
*
Vehicle
miR-29b mimic
Control mimic

Supp Fig 5
