# Supplementary material for: MicroRNA mimicry blocks pulmonary fibrosis
Source: EMBO Mol Med. 2014 Sep 19;6(10):1347–56. doi: 10.15252/emmm.201303604 (PMC4287936; doi:10.15252/emmm.201303604)
Supplement: Supplementary file 2 [file emmm0006-1347-sd2.pdf]

EMBO Molecular Medicine

# MicroRNA mimicry blocks pulmonary fibrosis

Rusty L. Montgomery, Guoying Yu, Paul A. Latimer, Christianna Stack, Kathryn Robinson, Christina M. Dalby, Naftali Kaminski, Eva van Rooij

*Corresponding authors: Eva van Rooij, Hubrecht Institute and Naftali Kaminski, Yale School of Medicine*

---

**Review timeline:**

| | |
|---|---|
| Submission date: | 25 October 2013 |
| Editorial Decision: | 30 November 2013 |
| Revision received: | 12 June 2014 |
| Editorial Decision: | 10 July 2014 |
| Accepted: | 20 August 2014 |

---

## Transaction Report:

(Note: With the exception of the correction of typographical or spelling errors that could be a source of ambiguity, letters and reports are not edited. The original formatting of letters and referee reports may not be reflected in this compilation.)

*Editor: Céline Carret*

1st Editorial Decision 30 November 2013

---

Thank you for the submission of your manuscript to EMBO Molecular Medicine. We have now heard back from the three referees whom we asked to evaluate your article. Although the referees find the study to be of potential interest, they also raise a number of concerns that need to be thoroughly addressed in a major revision of your work.

As you can see from the reports below, all three referees find the topic very interesting but while referee 1 is rather supportive of publication, referees 2 and 3 are more reserved and raised overlapping concerns. These two referees request additional experiments to better support the conclusions. In particular, they point towards the timing of the effects that should be better documented, the clinical relevance that needs to be developed, the mechanistic insights that are too limited (and referee 3 suggests to investigate the cell type targeted by the miRNA mimic), and finally better discussion and explanations that should be expanded.

Given these evaluations, I would like to give you the opportunity to revise your manuscript, with the understanding that the referees' concerns must be fully addressed and that acceptance of the manuscript would entail a second round of review. However, please note that that it is our journal's policy to allow only a single round of revision, and that acceptance or rejection of the manuscript will therefore depend on the completeness of your response and the satisfaction of the referees with it.

EMBO Molecular Medicine has a "scooping protection" policy, whereby similar findings that are published by others during review or revision are not a criterion for rejection. Should you decide to submit a revised version, I do ask that you get in touch after three months if you have not completed it, to update us on the status.

Please also contact us as soon as possible if similar work is published elsewhere. If other work is published we may not be able to extend the revision period beyond three months.

I look forward to receiving your revised manuscript.

***** Reviewer's comments *****

Referee #1 (Comments on Novelty/Model System):

The manuscript has the most innovative component in the use of microRNA mimicry for increasing miRNAs in target organs; the approach that has been used successfully so far for modulating miRNAs in target tissues is through antisense approach.

The work is therefore potentially important. I raised some criticisms on issues related to the choice of miR-29b and other issues related to the targeted effects of this approach as related to off-targeted ones.

Referee #1 (Remarks):

This is an interesting manuscript, in which miR29b is overexpressed in vivo through miR-mimicry. This is one of the first manuscripts in which this approach has been used and thus it is potentially relevant.

I have the following criticisms:

1. The authors should explain why they used microRNA 29b instead of miR-29 a or c. Is there a specific reason?

2. Why there is a significant uptake only in the liver and not in the heart? Is there a possible explanation?

3. miRNA-29 controls macrophage response: the authors should try to determine the effects of miR-29 mimicry on macrophage activation, which is fundamental for fibrosis. They have done experiments in which the effects of miR-29 on inflammatory cell counts are measured. However, it would be most appropriate to show these effects also in macrophage activation assays in vitro.

4. The authors impute the effects of miRNA mimicry on fibroblast collagen production. However, other cells can be implicated, since miRNA-29 is ubiquitously expressed. Is there a direct effect on alveolar cells (beside the previous question related to macrophages)? The histology sections show a remarkable effect but it is somehow hard to distinguish the different components of the alveolar parenchyma.

Referee #2 (Comments on Novelty/Model System):

This article represents the first description of the therapeutic potential of using miRNA mimics to therapeutically increase miR-29b for the treatment of pulmonary fibrosis in an animal model of lung fibrosis. The novelty and medical impact of this study is potentially high. I have some technical concerns pertaining to the timing of the administration of these agents in this model. Further experiments using a therapeutic dosing regime during the fibrotic phase of this model are needed in order to support the main conclusions of this article.

Referee #2 (Remarks):

This article by Montgomery and colleagues represents the first description of the therapeutic potential of using miRNA mimics to therapeutically increase miR-29b for the treatment of pulmonary fibrosis in an animal model of lung fibrosis. The novelty and medical impact of this study is therefore potentially high but there are some issues with study design which dampen overall enthusiasm.

1. The reviewer is not sure why the authors chose to only assess hydroxyproline levels in the left lung (although figure states rt?). Bleomycin-induced fibrosis is very patchy and the gold-standard is to measure total lung collagen levels in all lobes in order to avoid sampling bias. This may explain why the increase in lung collagen in response to bleomycin injury is very low compared with the published literature. A two-fold induction is generally expected in this model. The reviewer is also somewhat surprised that the difference in collagen levels for bleo + miR-29b mimic versus bleo + control mimic is statistically significant (as illustrated by the star in the graph Figure 2D)? The error bars look like they overlap?

2. The data presented in Figure 2D (and to a lesser degree Fig 2C) is critical in terms of supporting a role for an anti-fibrotic effect of miR-29b administration in the bleomycin model. From the information provided it is not clear at which time point the miR-29b mimic was administered (3 or 10 days?) in this particular experiment. This needs to be clearly stated since a 3 day dosing regime would be considered very early and would represent the peak of the inflammatory response in this model. This observation will require confirmation in experiments where the miR-29b mimic is given during the fibrotic phase (from 7-10 days onwards). A 14 day time point for assessing the impact on fibrosis might be too early, the authors should therefore consider dosing at 7 days and sacrificing mice at 21 days to determine the role of this miR-29b mimic as a potential anti-fibrotic agent in this particular model. These confirmatory studies are particularly critical in light of the fact that the miR-29b mimic impacts on inflammation in this model (Figure 2H). Since blunting inflammatory cell recruitment is well-recognised to impact on the subsequent fibrotic response in the bleomycin model, the authors will need to address whether all the effects of miR-29b are indeed mediated by blunting inflammation rather than by influencing ECM gene expression directly during the fibrotic phase.

3. Figure 3A. The data presented in this Figure suggests that only Col1A1 gene expression is statistically increased following bleomycin injury. This is contradictory to the statement in the manuscript describing this figure where it is stated that there is a pronounced increase in both Col1A1 and Col3a1 gene expression. The authors will need to reword this sentence as it might be considered misleading as currently stated.

4. qPCR data - the housekeeping genes used for normalization are not listed.

5. In general, the information provided in this article is very much on the minimalist side. A more in depth final discussion paragraph bringing together current thinking regarding the role and the therapeutic potential of multiple miR therapeutic approaches in this disease setting would be helpful for the broad readership of this journal.

Referee #3 (Comments on Novelty/Model System):

The experiments are technically sound. They need to supplemented with further experiments to be convincing. The therapeutic approach is novelty and quite exciting. The author's need to perform additional experiments to establish biological relevance of this therapeutic approach.

Referee #3 (Remarks):

General Comment:

In this paper the author's report the efficacy of using a synthetic microRNA (miRNA) as a mimic to restore miRNA function as a treatment approach for pulmonary fibrosis. In a series of in vitro and mouse experiments they show the efficacy of a synthetically produced analog of miR29, a known regulator of extracellular matrix protein expression, in decreasing collagen expression in vitro and in protecting against bleomycin induced lung fibrosis. Targeting miRNA expression is an exciting and evolving area of therapeutics because it provides the potential of impacting expression of multiple genes simultaneously involved in a particular disease. Most reports to date have focused on inhibiting miRNAs and less has been reported on the efficacy of increasing the function of miRNAs. In this light, the data of Montogomery et al. is novel and could provide compelling proof of principle of the therapeutic approach of miRNA molecular mimicry. The data presented in this paper are interesting in that they report efficacy in the bleomycin lung fibrosis modes. However, the data incomplete in that the mouse data needs to be supplemented to be more convincing. In addition data on the putative efficacy mechanism is lacking, (i.e. which cell type is targeted in vivo, timing of effect, the relationship between innate immune and anti-fibrotic effects). Lastly data which would support biological relevance of this approach is rather limited.

Specific Comments:

1. The authors use a fibroblast cell line (NIH 3T) for their initial in vitro experiments to show that a miR29 mimic inhibits collagen expression in a dose dependent fashion. These data would be enhanced if the same data could be demonstrated in primary lung fibroblasts, both control and from patients with IPF. Demonstration of effects on protein expression would also improve these data.

2. The mouse experiments are suggestive of efficacy and the data on miR29 expression in whole organ lysates after IV injection of miR29 are convincing. It would be informative to asses which cells in the lung express miR29 after IV delivery. Demonstrating expression of miR29 in lung fibroblasts is a piece of data that is not provided and is crucial if one is to establish the miR29 is working via a fibroblast specific mechanism as suggested by their in vitro experiments.

3. Lung histology showing less fibrosis with miR29 is limited to one small cross-sectional piece of lung and is not particularly convincing since the bleomycin induced lung fibrosis is known to be patchy. A more compelling piece of evidence supplementing the hydroxyproline data would be useful.

4. The data on cytokines is not well explained. Though there is a clear decrease with miR29 treatment, it is not reported at what time point in the experiment BALs were performed. In addition the relevance of this finding is not well explained in the context of a fibroblast specific mechanism. Does miR29 regulate gene expression in innate immune cells? It would be useful to show levels of TGF- and CTGF in addition to IGF-1, both of which are produced by fibroblasts and important in the development of lung fibrosis.

5. While the authors clearly show that miR29 expression is decreased in IPF patients, no data is provided on whether miR29 mimicry is effective in decreasing matrix expression in fibroblasts from IPF patients.

6. It is not clear when miR29 was infused in the data that is shown. Was there a difference in efficacy with respect to timing of the infusion, i.e. day 3 or 10 after bleomycin instillation?

1st Revision - authors' response 12 June 2014

**Answers to reviewers**

Comments by editor:

As you can see from the reports below, all three referees find the topic very interesting but while referee 1 is rather supportive of publication, referees 2 and 3 are more reserved and raised overlapping concerns. These two referees request additional experiments to better support the conclusions. In particular, they point towards the timing of the effects that should be better documented, the clinical relevance that needs to be developed, the mechanistic insights that are too limited (and referee 3 suggests to investigate the cell type targeted by the miRNA mimic), and finally better discussion and explanations that should be expanded.

AU: We are glad all reviewers find our manuscript very interesting. In this updated version we were able to answer most points raised by the reviewers and extended our explanations and discussion.

Referee #1 (Comments on Novelty/Model System):
The manuscript has the most innovative component in the use of microRNA mimicry for increasing miRNAs in target organs; the approach that has been used successfully so far for modulating miRNAs in target tissues is through antisense approach.
The work is therefore potentially important. I raised some criticisms on issues related to the choice of miR-29b and other issues related to the targeted effects of this approach as related to off-targeted ones.

AU: We are glad the reviewer thinks are work is potentially important and we answered the criticisms that were raised.

Referee #1 (Remarks):
This is an interesting manuscript, in which miR29b is overexpressed in vivo through miR-mimicry. This is one of the first manuscripts in which this approach has been used and thus it is potentially relevant.
I have the following criticisms:

1. The authors should explain why they used microRNA 29b instead of miR-29 a or c. Is there a specific reason?

AU: We apologize for being unclear. miR-29b is consistently the most down-regulated miR-29 family member across multiple organ fibrosis. Given miR-29a, -b, and –c all have the same seed region, we synthesized one miR-29 mimic for use in fibrotic studies, and chose miR-29b.

2. Why there is a significant uptake only in the liver and not in the heart? Is there a possible explanation?

AU: We apologize for not explaining this better. We improved the description of tissue distribution in the current version of the manuscript to rule out any confusion about liver targeting. This mimic shows strong delivery to the lung,

which at this time is through unknown mechanisms.

3. miRNA-29 controls macrophage response: the authors should try to determine the effects of miR-29 mimicry on macrophage activation, which is fundamental for fibrosis. They have done experiments in which the effects of miR-29 on inflammatory cell counts are measured. However, it would be most appropriate to show these effects also in macrophage activation assays in vitro.

AU: We thank the reviewers for this comment. We transfected miR-29b mimic and control into macrophage cells, RAW 264.7 from Sigma, and harvested the supernatants at time point 24 and 48 hours after transfection of the cells. IFN-r, IL-1B, IL-2, IL-4, IL-5, IL-6, KC, IL-10, IL-12P70, and TNF-a were determined, however we only found KC marginally changed.
We also did not see an alteration of Tgfb1, Ctgf, FGF1, PDGF on the mRNA level, but did see the significant change of expression of Csf3, Igf1 and KC by qRT-PCR analysis.

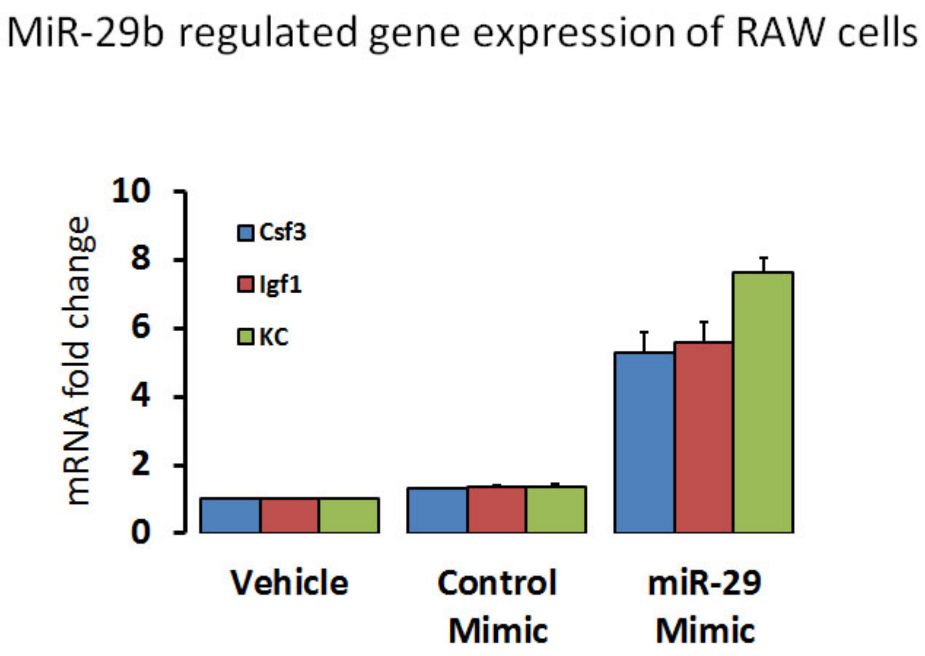


We have added this to the text and added the figure above to the supplemental data as well.

4. The authors impute the effects of miRNA mimicry on fibroblast collagen production. However, other cells can be implicated, since miRNA-29 is ubiquitously expressed. Is there a direct effect on alveolar cells (beside the previous question related to macrophages)? The histology sections show a remarkable effect but it is somehow hard to distinguish the different components of the alveolar parenchyma.

AU: We thank the reviewer for this point. While we believe the effects of miR-29 mimicry are mainly due to regulation of fibroblast collagen production, we agree that we cannot rule out the contribution of additional cell-types that

produce collagen.  To look into this further, we studied the effect of miR-29 mimicry in primary human IPF fibroblasts and an alveolar epithelial cell line after TGF-β treatment.  In both cases, miR-29 mimicry significantly blunted the collagen response.  This was seen in TGF-β treated cells, as well as in baseline conditions.  This data suggests miR-29 mimicry can work via multiple collagen producing cells where the mimic delivers and we have amended the text to address thisand added in the data shown below:

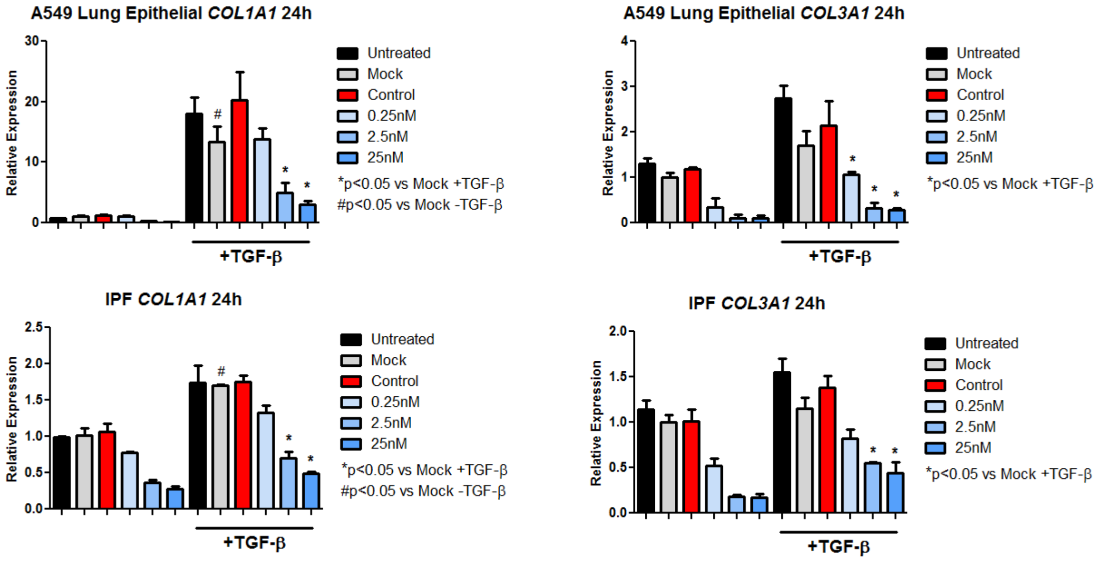


We also looked at THP-1 cells after miR-29 mimicry treatment, to see if collagen expression is altered in these cells.  However, we could not detect any collagen expression in these cells in vitro.

Referee #2 (Comments on Novelty/Model System):
This article represents the first description of the therapeutic potential of using miRNA mimics to therapeutically increase miR-29b for the treatment of pulmonary fibrosis in an animal model of lung fibrosis. The novelty and medical impact of this study is potentially high. I have some technical concerns pertaining to the timing of the administration of these agents in this model. Further experiments using a therapeutic dosing regime during the fibrotic phase of this model are needed in order to support the main conclusions of this article.

AU: Thank you for saying that the medical impact of our study is potentially high. We have now explained the dosing regimen better and added in data using miR-29b mimic in a therapeutic dosing regime.

Referee #2 (Remarks):
This article by Montgomery and colleagues represents the first description of the therapeutic potential of using miRNA mimics to therapeutically increase miR-29b for the treatment of pulmonary fibrosis in an animal model of lung fibrosis. The novelty and medical impact of this study is therefore potentially high but there are some issues with study design which dampen overall enthusiasm.

1. The reviewer is not sure why the authors chose to only assess hydroxyproline levels in the left lung (although figure states rt?). Bleomycin-induced fibrosis is very patchy and the gold-standard is to measure total lung collagen levels in all lobes in order to avoid sampling bias. This may explain why the increase in lung collagen in response to bleomycin injury is very low compared with the published literature. A two-fold induction is generally expected in this model. The reviewer is also somewhat surprised that the difference in collagen levels for bleo + miR-29b mimic versus bleo + control mimic is statistically significant (as illustrated by the star in the graph Figure 2D)? The error bars look like they overlap?

AU: We thank the reviewer for noticing this error and apologize for that. Indeed, we used only right lung for all hydroxyproline studies and used the entire right lung to address any patchiness associated with the model. We chose to use the right lung in every mouse for hydroxyproline for consistency and use the left lung for additional molecular analyses (i.e. real-time or histology). As for the significance, we have an n=10 for each group in the hydroxyproline studies, so while there might be a slight overlap, the study was powered well to reach statistical significance. Regarding the effect size, the increase in hydroxyproline is the consistent effect we see using this dose of bleomycin. It is important to note that different effects are seen with different sources of bleomycin and at different doses.

2. The data presented in Figure 2D (and to a lesser degree Fig 2C) is critical in terms of supporting a role for an anti-fibrotic effect of miR-29b administration in the bleomycin model. From the information provided it is not clear at which time point the miR-29b mimic was administered (3 or 10 days?) in this particular experiment. This needs to be clearly stated since a 3 day dosing regime would be considered very early and would represent the peak of the inflammatory response in this model. This observation will require confirmation in experiments where the miR-29b mimic is given during the fibrotic phase (from 7-10 days onwards). A 14 day time point for assessing the impact on fibrosis might be too early, the authors should therefore consider dosing at 7 days and sacrificing mice at 21 days to determine the role of this miR-29b mimic as a potential anti-fibrotic agent in this particular model. These confirmatory studies are particularly critical in light of the fact that the miR-29b mimic impacts on inflammation in this model (Figure 2H). Since blunting inflammatory cell recruitment is well-recognized to impact on the subsequent fibrotic response in the bleomycin model, the authors will need to address whether all the effects of miR-29b are indeed mediated by blunting inflammation rather than by influencing ECM gene expression directly during the fibrotic phase.

AU: We apologize for the confusion, and have amended the text in the body as well as the methods to more clearly state mimic treatment were administered twice: at 3 days post-bleomycin and 10 days post-bleomycin. Furthermore, we agree that the data in the original manuscript is more about prevention. To address the therapeutic potential of miR-29 mimicry in

established pulmonary fibrosis, we administered the miR-29 mimic at days 10, 14 and 17 and collected lungs at day 21. As can be seen in the figure below, the repeated administration of miR-29b led to blunting of the increases in hydroxyproline (A), COL1A1 (B) and COL3A1 (C) as well as amelioration of the histological changes seen after bleomycin (D). We believe these results support the notion that miR-29 mimicry should be considered as a therapeutic option in the context of established fibrosis.  We have added this data to the manuscript as well.

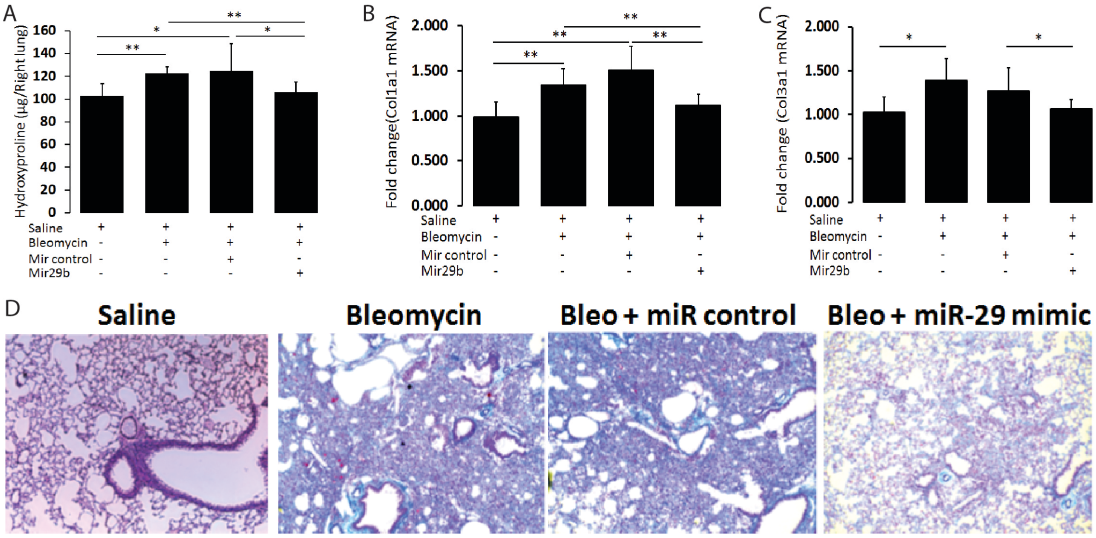


3. Figure 3A. The data presented in this Figure suggests that only Col1A1 gene expression is statistically increased following bleomycin injury. This is contradictory to the statement in the manuscript describing this figure where it is stated that there is a pronounced increase in both Col1A1 and Col3a1 gene expression. The authors will need to reword this sentence as it might be considered misleading as currently stated.

AU: We apologize for the incorrect statement and confusion.  We have corrected the text to reflect the figure.  Thank you for this.

4. qPCR data - the housekeeping genes used for normalization are not listed.

AU: We thank the reviewer for catching this.  We have added this to the methods.

5. In general, the information provided in this article is very much on the minimalist side. A more in depth final discussion paragraph bringing together current thinking regarding the role and the therapeutic potential of multiple miR therapeutic approaches in this disease setting would be helpful for the broad readership of this journal.

AU: We thank the reviewer for the suggestion and have incorporated a more in depth final discussion.

Referee #3 (Comments on Novelty/Model System):

The experiments are technically sound. They need to supplemented with further experiments to be convincing. The therapeutic approach is novelty and quite exciting. The author's need to perform additional experiments to establish biological relevance of this therapeutic approach.

AU: We are glad the reviewer thinks our work is novel and quite exciting. We have now added in more experiments on cell type contribution and supplemented the data using miR-29b mimic in a therapeutic dosing regime.

Referee #3 (Remarks):

General Comment:
In this paper the author's report the efficacy of using a synthetic microRNA (miRNA) as a mimic to restore miRNA function as a treatment approach for pulmonary fibrosis. In a series of in vitro and mouse experiments they show the efficacy of a synthetically produced analog of miR29, a known regulator of extracellular matrix protein expression, in decreasing collagen expression in vitro and in protecting against bleomycin induced lung fibrosis. Targeting miRNA expression is an exciting and evolving area of therapeutics because it provides the potential of impacting expression of multiple genes simultaneously involved in a particular disease. Most reports to date have focused on inhibiting miRNAs and less has been reported on the efficacy of increasing the function of miRNAs. In this light, the data of Montogomery et al. is novel and could provide compelling proof of principle of the therapeutic approach of miRNA molecular mimicry. The data presented in this paper are interesting in that they report efficacy in the bleomycin lung fibrosis modes. However, the data incomplete in that the mouse data needs to be supplemented to be more convincing. In addition data on the putative efficacy mechanism is lacking, (i.e. which cell type is targeted in vivo, timing of effect, the relationship between innate immune and anti-fibrotic effects). Lastly data which would support biological relevance of this approach is rather limited.

AU: We are glad the reviewer thinks that our data our novel and interesting and in this updated version we attempted to make the data even more compelling and convincing by adding data on cellular contribution and therapeutic dosing in vivo.

Specific Comments:
1. The authors use a fibroblast cell line (NIH 3T) for their initial in vitro experiments to show that a miR29 mimic inhibits collagen expression in a dose dependent fashion. These data would be enhanced if the same data could be demonstrated in primary lung fibroblasts, both control and from patients with IPF. Demonstration of effects on protein expression would also improve these data.

AU: We thank the reviewer for this point and looked at miR-29 mimicry effects in IPF fibroblasts with and without TGF-β stimulation. miR-29 mimicry treatment showed robust collagen regulation in baseline as well as TGF-β treated primary IPF fibroblasts. The figure below shows effects on Col1a1 and Col3a1 24 hours post transfection. 48 hours looks the same as well. We

attempted collagen westerns but have been unsuccessful in getting them to work technically from tissue culture.

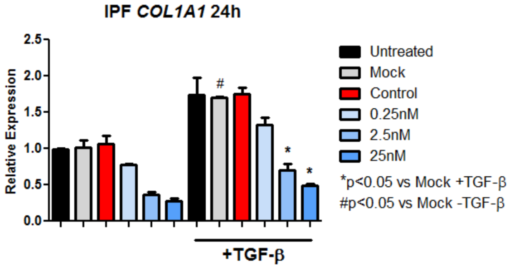


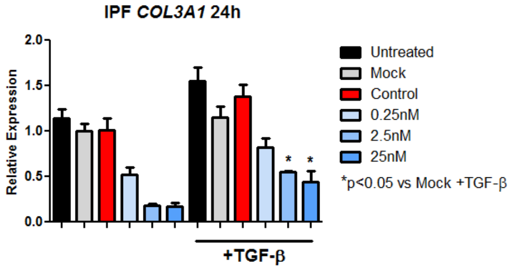


2. The mouse experiments are suggestive of efficacy and the data on miR29 expression in whole organ lysates after IV injection of miR29 are convincing. It would be informative to asses which cells in the lung express miR29 after IV delivery. Demonstrating expression of miR29 in lung fibroblasts is a piece of data that is not provided and is crucial if one is to establish the miR29 is working via a fibroblast specific mechanism as suggested by their in vitro experiments.

AU: We thank the reviewer for this point and are attempting to address this currently, however it is proving to be quite challenging via ISH or cell-fractionation techniques.  However, we have looked in additional cell types that express collagens in the lung, alveolar epithelial cells.  miR-29 mimic treatment to these cells does indeed result in a blunting of TGF-β induced collagen expression, suggesting the miR-29 mimicry in vivo can be acting via multiple cell-types.  We don't believe miR-29 has to be acting via a fibroblast-specific mechanism, rather, one where miR-29 can exert its effects on multiple cell types responsible for the fibrotic response. Below is data on alveolar epithelial cells:

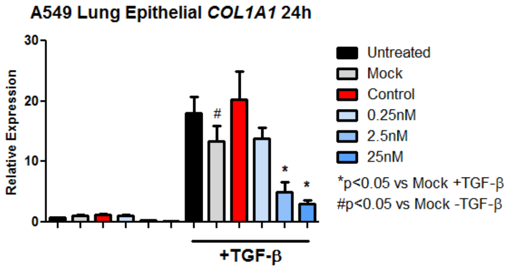


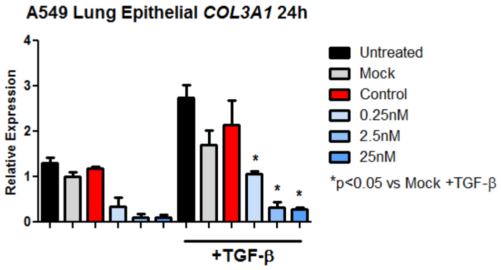


3. Lung histology showing less fibrosis with miR29 is limited to one small cross-sectional piece of lung and is not particularly convincing since the bleomycin induced lung fibrosis is known to be patchy. A more compelling piece of evidence supplementing the hydroxyproline data would be useful.

AU: We agree with the reviewer on the patchiness of bleomycin-induced fibrosis and the histology images while striking are illustrative, However we provide histology, hydroxyproline and measures of collagen mRNA and all of them demonstrate the same results.  More importantly, we did repeat the study in a therapeutic treatment paradigm.  In doing so, we saw similar results, where hydroxyproline content in the whole right lobe was significantly reduced with miR-29 treatment. We believe the hydroxyproline data, coupled

with the real-time and histological data, from 2 different treatment studies, supports the notion of miR-29 regulating pulmonary fibrosis.

4. The data on cytokines is not well explained. Though there is a clear decrease with miR29 treatment, it is not reported at what time point in the experiment BALs were performed. In addition the relevance of this finding is not well explained in the context of a fibroblast specific mechanism. Does miR29 regulate gene expression in innate immune cells? It would be useful to show levels of TGF-β and CTGF in addition to IGF-1, both of which are produced by fibroblasts and important in the development of lung fibrosis.

AU: WE apologize for not being more clear. BAL was done when the mice were harvested on day 14 in the preventive protocol. To address the question of effect of miR-29 on immune cells we transfected miR-29b mimic and control into macrophage cells, RAW 264.7 from Sigma, and harvested the supernatants at time point 24 and 48 hours after transfection and the cells. IFN-r, IL-1B, IL-2, IL-4, IL-5, IL-6, KC, IL-10, IL-12P70, and TNF-a were determined, the only finding was KC changed marginally. We were also unable to detect an alteration of Tgfb1, Ctgf, FGF1, PDGF on the mRNA level, but did see the significant change of expression of Csf3, Igf1 and Kc by qRT-PCR analysis.

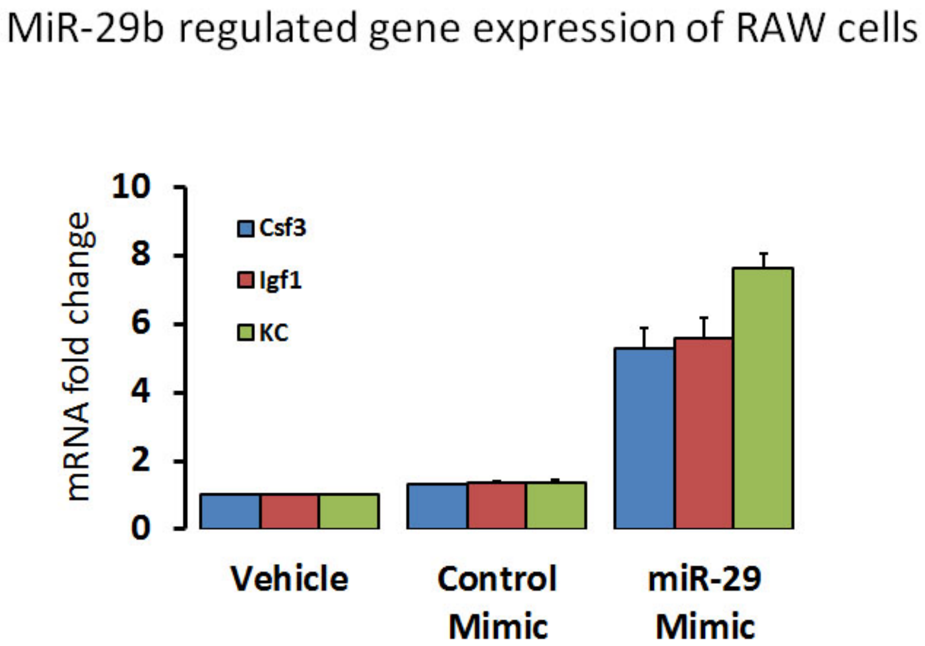


We have added this to the text and supplemental data as well.

5. While the authors clearly show that miR29 expression is decreased in IPF patients, no data is provided on whether miR29 mimicry is effective in decreasing matrix expression in fibroblasts from IPF patients.

AU: As mentioned in response to question 1, we did look at miR-29 mimicry effects in IPF fibroblasts with and without TGF-β stimulation. miR-29 mimicry treatment showed robust collagen regulation in baseline as well as TGF-β

treated primary IPF fibroblasts.  The figure below shows effects on Col1a1 and Col3a1 24 hours post transfection.  48 hours looks the same as well.

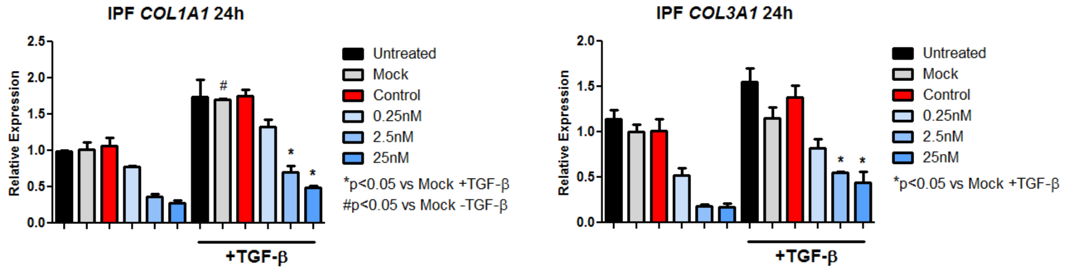


6. It is not clear when miR29 was infused in the data that is shown. Was there a difference in efficacy with respect to timing of the infusion, i.e. day 3 or 10 after bleomycin instillation?

AU: We apologize for the confusion.  We have amended the text to more accurately demonstrate the dosing paradigm used. In the current version of the manuscript have two administration regimens: Preventive in which miR-29 mimicry is administered at day 3 after bleomycin, with a repeat dose at day 10 and lungs are harvested at day 14 and therapeutic in which miR-29 is administered at day 10 and repeat does are given at day 14 and 17 and the lungs are harvested at day 21.

2nd Editorial Decision 10 July 2014

Thank you for the submission of your revised manuscript to EMBO Molecular Medicine. We have now received the enclosed reports from the referees that were asked to re-assess it. As you will see the reviewers are now globally supportive and I am pleased to inform you that we will be able to accept your manuscript pending the following final amendments:

As you can see, while both referees are overall satisfied, they both still mentioned a few limitations that we would like you to discuss briefly in the Results and Discussion part of your article.

Please submit your revised manuscript within two weeks.

I look forward to reading a new revised version of your manuscript as soon as possible.

***** Reviewer's comments *****

Referee #2 (Remarks):

This is a much improved version of the original article submitted and the findings are likely to be of great interest to the fibrosis community. The inclusion of new data from therapeutic dosing experiments during the fibrotic phase in the bleomycin model greatly strengthens the proposed mechanism of action of mir29 mimics and the potential clinical feasibility of exploring this approach in the context of fibrotic lung disease. This reviewer is still not completely convinced that the method for assessing lung collagen accumulation in this model is ideal but is prepaperd to accept that the body of evidence presented in this article supports the major conclusions. This reviewer has some reservations regarding the usefulness of the new A549 data and would suggest that the authors consider either removing this data altogether or expanding the Discussion section highlighting that this cell type is not considered a very reliable cell type in the context of human fibrotic lung disease. The issue as to whether type II cells are likely to represent a major source of collagen in the context of lung fibrosis is also currently unproven.

Referee #3 (Comments on Novelty/Model System):

The bleomycin model of lung fibrosis poorly capitulates IPF. It would strengthen the translatability of the approach of miRNA mimicry if the authors replicated their data in a 2nd model of lung fibrosis (e.g. TGF-b overexpression, asbestos).

Referee #3 (Remarks):

MicroRNA mimicry blocks pulmonary fibrosis
General Comment:
In this revised paper the author's report the efficacy of using a synthetic microRNA (miRNA) as a mimic to restore miRNA function as a treatment approach for pulmonary fibrosis. In the revision they have added new data that addresses most of my previous critiques (specifics are below) and strengthens their findings. Even though the authors have not addressed every concern in my previous critique and the mechanism by which miRNA mimicry works is not fully elucidated, they do make a convincing case for the potential of miRNA mimicry as a therapeutic option for lung diseases.

Previous Comments:

1. The authors use a fibroblast cell line (NIH 3T) for their initial in vitro experiments to show that a miR29 mimic inhibits collagen expression in a dose dependent fashion. These data would be enhanced if the same data could be demonstrated in primary lung fibroblasts, both control and from

patients with IPF. Demonstration of effects on protein expression would also improve these data. Data on IPF fibroblasts addresses this concern, though no data on protein expression is included.

2. The mouse experiments are suggestive of efficacy and the data on miR29 expression in whole organ lysates after IV injection of miR29 are convincing. It would be informative to asses which cells in the lung express miR29 after IV delivery. Demonstrating expression of miR29 in lung fibroblasts is a piece of data that is not provided and is crucial if one is to establish the miR29 is working via a fibroblast specific mechanism as suggested by their in vitro experiments. Though they were not able to show any in vivo data, the experiments with an epithelial cell line and IPF fibroblasts addresses this concern to a degree.

3. Lung histology showing less fibrosis with miR29 is limited to one small cross-sectional piece of lung and is not particularly convincing since the bleomycin induced lung fibrosis is known to be patchy. A more compelling piece of evidence supplementing the hydroxyproline data would be useful. This is now addressed.

4. The data on cytokines is not well explained. Though there is a clear decrease with miR29 treatment, it is not reported at what time point in the experiment BALs were performed. In addition the relevance of this finding is not well explained in the context of a fibroblast specific mechanism. Does miR29 regulate gene expression in innate immune cells? It would be useful to show levels of TGF- and CTGF in addition to IGF-1, both of which are produced by fibroblasts and important in the development of lung fibrosis. This is partially addressed with the experiments in macrophage cell lines.

5. While the authors clearly show that miR29 expression is decreased in IPF patients, no data is provided on whether miR29 mimicry is effective in decreasing matrix expression in fibroblasts from IPF patients. The data with IPF fibroblasts addresses this concern.

6. It is not clear when miR29 was infused in the data that is shown. Was there a difference in efficacy with respect to timing of the infusion, i.e. day 3 or 10 after bleomycin instillation? The additional experiments with the infusion beginning at day 10 address this concern.

© EMBO
